# Management of Infectious Lymphadenitis in Children

**DOI:** 10.3390/children8100860

**Published:** 2021-09-27

**Authors:** Francesco Pecora, Luciana Abate, Sara Scavone, Irene Petrucci, Federico Costa, Caterina Caminiti, Alberto Argentiero, Susanna Esposito

**Affiliations:** 1 Paediatric Clinic, Pietro Barilla Children’s Hospital, Department of Medicine and Surgery, University of Parma, via Gramsci 14, 43126 Parma, Italy; cescopec@hotmail.it (F.P.); luciana.abate@studenti.unipr.it (L.A.); sara.scavone92@gmail.com (S.S.); petrucciirene94@gmail.com (I.P.); costa.fede95@gmail.com (F.C.); alberto.argentiero@unipr.it (A.A.); 2 Research an Innovation Unit, University Hospital of Parma, via Gramsci 14, 43126 Parma, Italy; ccaminiti@ao.pr.it

**Keywords:** lymphadenitis, *Bartonella henselae*, mycobacterium avium complex, children

## Abstract

Lymphadenopathy is an irregularity in the size and texture of the lymph nodes, which is quite common in childhood. When the enlargement of lymph nodes is caused by inflammatory and infectious processes, it is called lymphadenitis. The main objective of this manuscript is to summarize the common infectious etiologies and presentations of lymphadenitis in children providing a management guide for clinical practice. PubMed was used to search for all of the studies published up to April 2021 using keywords such as “lymphadenitis” and “children”. Literature analysis showed that the differential diagnosis for lymphadenitis in pediatrics is broad. Although lymph node enlargement in children is usually benign and self-limited, it is important to exclude malignant etiology. In most cases, history and physical examination allow to identify the correct diagnosis and start a proper treatment with a prompt resolution of the lymphadenopathy. However, particularly in the case of persistent lymphadenitis, determining the cause of lymph node enlargement may be difficult, and the exact etiology may not be identified despite extensive investigations. Further studies should develop and validate an algorithm to assist pediatricians in the diagnosis and timely treatment of lymphadenitis, suggesting situations in which a watchful waiting may be considered a safe approach, those in which empiric antibiotic therapy should be administered, and those requiring a timely diagnostic work-up.

## 1. Introduction

Lymphadenopathy is described as an irregularity in the size and texture of the lymph nodes. This condition is common in childhood, and it can be due to a variety of etiologies: the most prevalent causes are local or generalized infections, while autoimmune and neoplastic processes are less frequent causes [1,2]. The exact peak incidence in the pediatric population isn’t known, but many analyses demonstrate that 45–57% of apparently healthy children may have palpable lymph nodes at any time [3,4]. About 90% of children between the ages of 4 and 8 suffer from lymphadenopathy [5,6].

Lymph nodes in children are usually and physiologically bigger than in adolescents and adults; this happens because of the continuous exposure to new antigens [7,8,9,10]. The enlargement of lymph nodes caused by inflammatory and infectious processes is called lymphadenitis [7]. Inflammation and infection of the lymph nodes can occur not only with the increased size of the nodes, but they can also be associated with pain, skin changes, edema, fever, and/or purulent exudate [1].

In general, lymphadenitis can be distinguished as acute if it lasts up to 2 weeks; subacute if it lasts from 2 to 6 weeks; and chronic if it lasts more than 6 weeks. This classification is not really categorical, and some disease processes may fall into more than one category related to duration and presentation of symptoms. With regard to the etiology, acute lymphadenitis can be due to either viral or bacterial pathogens. Subacute lymphadenitis includes a larger group of potential etiologies. Instead, chronic lymphadenopathies are caused, in most cases, by a neoplastic process [1,3,11].

The main objective of this manuscript is to discuss the approach to diagnosis and management of infectious lymphadenitis in children, focusing on the characteristics of the main infectious agents involved in the pediatric age. PubMed was used to search for all of the studies published up to April 2021 using keywords such as “lymphadenitis” and “children”. The search was limited to articles published in English and providing evidence-based data.

## 2. Pathophysiology

Amongst secondary lymphoid organs, lymph nodes play an essential role [12]. Histologically, lymph nodes can be divided into two main areas: the medulla and the cortex [13,14]. While the medulla is made of a deep net of lymph-draining sinuses detached by medullary strings containing mainly plasma cells and secondarily macrophages and memory T-cells (with a not yet clearly understood function), the cortex can be considered the cornerstone of the lymph node. The cortex itself is also divided into the paracortex containing T-cells and an external B-cell area consisting of primary follicles and potential germinal centers after the antigen challenge has been fulfilled. The humoral responses take place in the B-cell follicles, whereas the paracortex is the region where lymphocytes circulating in the lymphatic vessels actually enter the lymph node and where interaction between T cells and dendritic cells occurs [13,14,15,16].

Particulate matter and antigens, either produced in different tissues or entered through epithelial tears, are rapidly directed into the lymphatic channels and therefore phagocytized by macrophages and other antigen-presenting cells [17]. After being phagocytized, foreign antigens become bound to major histocompatibility (MHC) proteins and are exposed to the surface of macrophages. Exogenous proteins are further bound to MHC class II molecules on the surface of dendritic cells [18]. The activation of T-helper lymphocytes requires a combination with other cell surface receptors and complex cellular signaling involving many secreted proteins such as but not limited to interleukins [19,20]. Furthermore, T-helper lymphocytes contribute to the activation of naïve B-lymphocytes, and dendritic cells can also directly activate memory B-lymphocytes. After that, B- and T-lymphocytes expand and replicate into the creation of a pool of lymphocytes capable of recognizing and binding the so considered threatening foreign protein. Finally, activated T-lymphocytes and macrophages intermediate in cellular signaling by releasing cytokines and many other proteins that induce chemotaxis of leukocytes and induce vascular permeability [21].

The variety of the symptoms associated with acute cervical lymphadenitis reflect the pathophysiologic series of events that occur secondary to infection: edema of the tissues, hyperplasia of the lymphocytes, leukocytes infiltration, and chemotaxis result in nodal enlargement [22]. On the other hand, the local release of cytokines and many other cellular signals induce not only vasodilation and capillary leak but also erythema and edema of the protective top skin, and lastly, tenderness as a result of the bloating of the nodal capsule [22].

## 3. Clinical History

Infections are the most common causes of lymphadenopathy. Clinical history should consider the patient’s age, the time course of the disease, associated symptoms, zoonosis, and previous travels [7].

In neonates, lymph nodes enlargement may express a congenital infection, mainly due to cytomegalovirus or *Toxoplasma gondii* [7]. In children between 3 and 5 years old, upper respiratory tract infections, pharyngitis, otitis, or conjunctivitis frequently cause reactive cervical or submandibular lymphadenopathy [1]. Sexually transmitted diseases are a possible cause of inguinal lymphadenopathy in adolescents [23].

Then, clinicians should ascertain the duration of the lymphadenopathy, the possible progression in size, site, and number of lymph nodes involved [1]. Acute enlargement is more suggestive of acute viral or bacterial infection, and it is usually unilateral in bacterial infections of the region drained by that lymph nodes [1]. Viral infections usually cause bilateral lymphadenopathy in the case of respiratory viruses (i.e., influenza, adenovirus) or generalized lymphadenopathy in the case of EBV or CMV infections [1].

Lymph node enlargement that solves in 2–3 weeks is considered acute lymphadenopathy, while if it lasts more than 4–6 weeks, it is considered chronic lymphadenopathy [24].

Associated symptoms such as hepatomegaly, persistent fever, weight loss, asthenia, sweats, cough or rash, joint pain, or recurrent infections may be useful data to make a correct diagnosis and to consider other possible causes of lymphadenopathy [23]. The most frequent noninfectious causes of lymphadenopathy are medications, malignancy, immunologic disorders, or Kawasaki disease [7,23].

Contact with animals or international travels should be investigated to direct diagnostic suspicion [7]. Table 1 summarizes the main zoonosis associated with acute lymphadenitis in the pediatric age.

## 4. Physical Examination

Palpation of the lymph nodes is an important component of a child’s examination, and the definition of abnormal lymph nodes depends on many features, including their size, location, and quality [23,25].

In childhood, a lymph node can be considered abnormal in size if it has a diameter larger than 10 mm in the cervical or axillary area, 15 mm in the inguinal region, 5 mm in the post-auricular area and in the epitrochlear area, and 1–2 mm in the supraclavicular area [25].

The location can direct the clinician to search for the possible sources of infection [25]. For example, axillary lymphadenitis can be related to cat-scratch disease, whereas cervical lymphadenitis can be related to streptococcal pharyngitis, and submandibular node enlargement could usually be due to oral infection. A palpable lymph node in the supraclavicular area is worrisome and need differential diagnosis with malignancy. Otherwise, inguinal enlarged lymph nodes can be an expression of sexually transmitted diseases or in neonates of congenital infections [23].

Nevertheless, localized lymphadenopathy is expression of infection of the node itself or from an infection in its drainage area (Table 2). On the contrary, generalized lymphadenopathy is caused by systemic disease [23].

Usually, soft lymph nodes, easily compressible and mobile on the underlying tissues, are benign [23]. On the other hand, malignances lymph nodes are characterized by hard (due to fibrosis), matted, and rubbery nodes [23].

## 5. Investigation

Investigation of lymphadenitis includes laboratory examinations, radiologic evaluation and fine-needle aspiration or excisional biopsy in selected cases.

Laboratory exams may be done in chronic and generalized lymphadenopathy or in the presence of acute localized lymphadenopathy that seems unresponsive to empirical antibiotic therapy or not self-limiting. A rapid test for the identification of *Streptococcus pyogenes* antigens in pharyngeal samples is almost always recommended [7]. A complete blood cell count with differential count, erythrocyte sedimentation rate, C-reactive protein, and liver enzymes are first level exams to evaluate a possible infection disease [24]. Altered blood values of serum lactate dehydrogenase, uric acid, or peripheral smear are associated with malignancy [24]. Other laboratory tests should be performed when specific infectious causes are suspected. In these cases, serology may be obtained to search the evidence of infection by EBV, HIV, CMV, Parvovirus B 19, Bartonella spp., Brucella spp., or *Toxoplasma gondii* [7]. In addition, interferon-gamma release assay or intradermal skin testing with PPD is performed when tuberculosis or *Mycobacterium avium* complex is suspected [6].

A chest radiograph is essential in case of chronic lymphadenopathy to study mediastinal widening due to lymphoma and sarcoidosis or to study mediastinal lymph node enlargement compressing the airway [7]. Furthermore, chest radiography may show hilar lymphadenomegaly and calcification due to tuberculosis [7]. Radiography of the neck is indicated to evaluate retropharyngeal space in case of consistent cervical and submandibular lumps [7].

Ultrasonography is a noninvasive imaging procedure that may be helpful in differentiating non-suppurative lymphadenopathy from suppurative lymphadenopathy, in estimating the size and monitoring it, and in identifying the fine echotexture of the lymph nodes [7,24,26]. Indeed, the presence of multiple enlarged lymph nodes in the same area may suggest mycobacteriosis, while bilateral lymph nodes are more typical of chronic non-specific lymphadenopathies. Colliquation inside the lump is observed almost exclusively in reactive lymphadenopathies [27].

Computed tomography (CT), especially with contrast, is more sensitive than ultrasonography to get anatomic details required before a surgical procedure. CT scan may provide additional anatomic information about nerves, blood vessels, and deep neck space to find a phlegmon or a retropharyngeal abscess and could be required when surgery is needed [7,24].

Although clinical or radiological features may drive to the most probable diagnosis, they can’t predict alone the correct one. For this reason, fine-needle aspiration of the lymph nodes content can be useful to determine the etiology of the lymphadenopathy. Indeed, the material can be studied with molecular assays like polymerase chain reaction (PCR) and cultures for aerobic and anaerobic bacteria, fungal, and mycobacterial organisms [28]. However, the potential formation of a fistulous tract as a complication in case of mycobacterial infection must be considered [24]. Moreover, the needle aspiration has a high false-negative rate in the case of malignancies [7,29].

**Figure 1:**
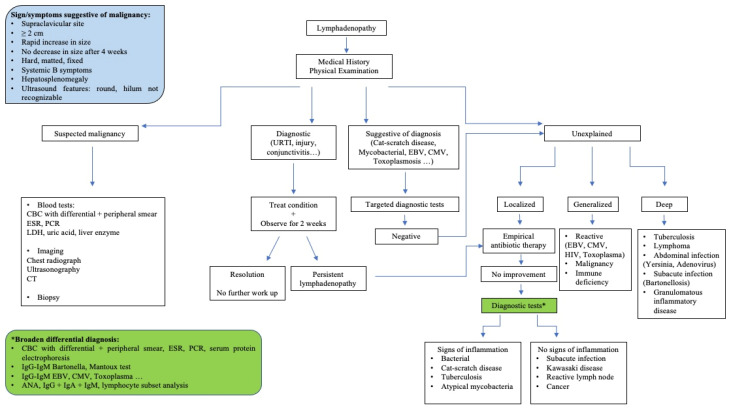
A proposed diagnostic and therapeutic approach to lymphadenitis in children. ANA, anti-nuclear antibodies; CBC, cell blood count; CT, computed tomography; CMV, cytomegalovirus; EBV, Epstein-Barr virus; ESR, erythrocyte sedimentation rate; HIV, human immunodeficiency virus; LDH, lactate dehydrogenase; PCR, C reactive protein.

Surgical management is indicated only when the suspicion of malignancy is high or when the lymph node is complicated by large colliquation or chronic draining sinus [6,30]. Definitive diagnosis is made on histology and microbiological investigation to search aerobic and anaerobic bacteria, fungal, and mycobacterial organisms through PCR and cultures of the piece of the lymph node excised [28,31]. Excisional biopsy is the treatment of choice for cervical lymphadenopathy caused by atypical mycobacteria [23]. Figure 1 shows a proposed diagnostic and therapeutic approach to lymphadenitis in children.

## 6. Acute Infectious Lymphadenitis

### 6.1. Acute Viral Lymphadenitis

In acute lymphadenitis of viral origin, lymph nodes involvement is typically bilateral and is frequently associated with upper respiratory tract infection. In most cases, cervical lymph nodes are involved [6,32]. They are usually tender, with a small enlargement, and the overlying skin does not appear erythematous. They rarely become fluctuant with abscess formation, and the disease process generally resolves spontaneously over 7–10 days [6,32]. The viruses that most frequently cause acute lymphadenitis associated with upper respiratory tract infection include rhinovirus, parainfluenza virus, influenza virus, respiratory syncytial virus, common coronaviruses, and adenovirus. In these cases, acute lymphadenitis is often associated with other signs and symptoms such as fever, ear pain, or pharyngitis with sore throat [7,32].

CMV and EBV are quite often responsible for the onset of acute generalized lymphadenitis, while rarer etiologies of acute generalized disease include mumps, measles, rubella, varicella, herpes simplex, human herpesvirus 6 (roseola), and coxsackie viruses [1,11,22,32,33]. Generalized lymphadenopathy is characterized by the involvement of two or more lymph nodes in noncontinuous regions of the body.

### 6.2. Acute Bacterial Lymphadenitis

Bacterial infections in preschool-age children usually appear as a sudden enlargement (frequently around 2–3 cm) of a solitary, tender, unilateral cervical lymph node [33]. Generally, submandibular lymph nodes are the most frequently involved, followed by upper cervical, submental, occipital, and ultimately, lower cervical nodes. According to Kelly et al., in children aged 1–4 years, *Staphylococcus aureus* or *Streptococcus pyogenes* (GABHS) are the main causes of acute unilateral cervical lymphadenitis [33,34,35]. Lymphadenitis caused by GABHS usually display in the child with unilateral submandibular or facial bloating, tenderness, and erythema but also with fever, pharyngitis, lack of appetite, and irritability. Anaerobic bacteria, including different species as *Peptococcus* sp., *Peptostreptococcus* sp. and *Bacteroides* sp, can cause acute lymphadenitis with odontostomatology issues as periodontal disease and dental caries in older children [1]. Less frequently, acute lymphadenitis can be provoked by uncommon bacteria such as *Pasteurella multocida* and *Francisella tularensis*, while other microorganisms such as *Streptococcus pneumoniae*, *Staphylococcus epidermidis*, group C and α-hemolytic streptococci, Gram-negative bacilli, and *Yersinia enterocolitica* are very rarely the etiology of the phenomenon [22].

Several cases of acute lymphadenitis caused by community-acquired methicillin-resistant *S. aureus* (CA-MRSA) have been recently reported [36,37,38]. Considering the increasing documented nasopharyngeal colonization by methicillin-resistant strains in healthy children that have recently been reported, the CA-MRSA will probably become the prevalent agent causing cervical lymphadenitis in children in the future [39,40].

## 7. Subacute Infectious Lymphadenitis

### 7.1. Nontuberculous Mycobacteria Lymphadenitis

Nontuberculous mycobacteria (NTM) are ubiquitous mycobacteria found in soil, water, food, animals, and other environmental sites [41,42]. Infection usually occurs by contact, aspiration, or inoculation, and no definitive evidence of person-to-person transmission of NTM exists. Although there are more than 130 recognized species, the majority of human NTM disease is caused mainly by the *Mycobacterium avium* complex (MAC). Other species causing infection in children are *M. fortuitum*, *M. lentiflavum*, *M. abscessus*, *M. kansasii*, *M. marinum*, *M. chelonae*, and *M. ulcerans* [41,42].

The incubation periods are variable. Based on the time required to achieve sufficient growth for identification, they are distinguished in “rapidly” or “slow-” growing mycobacteria. The most common species causing lymphadenitis are slowly growing species: MAC, *M. lentiflavum, M. kansasii*, and *M. fortuitum* [41,42].

In the immunocompetent child, NTM lymphadenitis is the most common manifestation, with the cervicofacial region predominantly being affected [43,44]. Several prospective surveillance studies reported an incidence of NTM lymphadenitis ranged from 0.8 to 3.5 per 100,000, with the highest incidence rates in children <4 years of age [45,46,47]. The most frequent sites of infection are submandibular and cervical lymph nodes, followed by the preauricular region [48]. Affected children usually present with a unilateral, subacute, and slowly enlarging lymph node in the absence of constitutional symptoms. Typically, an extension of disease beyond the local site is rare. The involved lymph node is initially firm, freely movable, and painless, and the skin is not erythematous. After several weeks, the lymph node undergoes rapid suppuration: the center of the node becomes fluctuant, and purple discoloration of the overlying skin occurs. Eventually, the nodes rupture and can form cutaneous fistulous tracts with the discharge of purulent material [49,50,51]. In most children with NTM infection, blood tests usually do not show an increase in the white blood cell count (WBC) or a significant increase in the inflammatory markers [52]. Ultrasonography may be useful in monitoring NTM lymphadenopathy and detecting signs of colliquation, and it often reveals more extensive disease than apparent on physical examination [53]. Definitive diagnosis of NTM lymphadenitis requires isolation of the mycobacterial specimen by culture or PCR testing [49,54,55,56].

Tuberculin skin test (TST) can be useful in the diagnostic work-up of children with a high clinical suspicion of NTM lymphadenitis, particularly when culture and PCR results are negative. Purified protein derivative (PPD) used in TST is a heterogeneous mixture of mycobacterial peptides, some of which are expressed by both *M. tuberculosis* and NTM [57]. Therefore, children with NTM lymphadenitis can have slightly positive TST results. In a study that enrolled 112 children with NTM cervicofacial lymphadenitis, Lindeboom et al. showed that at the optimal cut-off for a positive test (5 mm), TST had a sensitivity and specificity of 70% and 98%, respectively, and a positive predictive value and a negative predictive value of 98% and 64%, respectively [58]. Thus, TST could be helpful as a first step in the diagnostic analysis of cervicofacial lymphadenitis in children who have not received BCG vaccination and in whom *M. tuberculosis* infection is ruled out (normal chest radiograph and no history of exposure to tuberculosis). Interferon-gamma release assays (IGRAs), i.e., in vitro tests that rely on the detection of interferon-gamma secreted by memory T cells following stimulation with mycobacterial antigens, which show a high specificity for *M. tuberculosis*, can cause cross-reaction with several NTM species (*M. kansasii, M. marinum*, and *M. szulgai*) [57,59,60,61]. In a study in which were enrolled 73 children (28 with bacteriologically confirmed TB, 23 with bacteriologically confirmed NTM lymphadenitis, and 22 with other nonmycobacterial respiratory tract infections), Detjen et al. demonstrated the ability of IGRAs, when performed in addition to the TST, to distinguish positive TST result caused by NTM disease [62]. Therefore, in cases of children with a positive TST with a negative IGRA, the results suggest that an NTM is likely the cause of the infection [53,63].

Although the current evidence about the optimal management of NTM lymphadenitis is limited, the “gold standard” treatment is the complete surgical excision [64,65]. This procedure is both curative and diagnostic since it includes the opportunity to obtain samples for histological analysis and microbiological confirmation. Table 3 summarizes the main studies on the management of NTM lymphadenitis in pediatric age [46,48,56,57,66,67,68,69,70].

Lindeboom et al. have conducted three randomized controlled trials (RCTs) investigating the treatment and management of NTM lymphadenitis [66,67,68,69]. In the first of these three RCT studies, 100 immunocompetent children with NTM lymphadenitis were randomly assigned to undergo surgical excision of the involved lymph nodes or to receive antibiotic therapy with clarithromycin and rifabutin for at least 12 weeks [66]. Surgical excision was more effective than antibiotic therapy, resulting in higher short-term cure rates (96% vs. 66%). Postoperative weakness of the marginal branch of the facial nerve as a complication of surgery was observed in seven patients (14%), but only in one patient was permanent [66]. Furthermore, using the revised and quantitative OSAS (Observer Scar Assessment Scale) scoring, the authors showed a significant better esthetic outcome after surgical treatment than after antibiotic treatment [67]. In another RCT performed by Lindeboom et al., 50 children with an advanced stage of cervicofacial NTM lymphadenitis (characterized by the fluctuation of the lymph node and discoloration of the skin) were enrolled and randomized to receive antibiotic therapy or to be given a wait-and-see approach [68]. No significant difference in the time to resolution of NTM cervicofacial lymphadenitis was revealed when comparing clarithromycin and rifabutin antibiotic treatment with a wait-and-see policy (36 weeks vs. 40 weeks median time) [68]. In a third RCT, 50 children with a culture-confirmed diagnosis of cervicofacial NTM lymphadenitis in the advanced stage were randomized to surgical excision of the involved lymph nodes or to surgical curettage [69]. Although both surgical strategies led to cure, the resolution of the disease was delayed in most of the children treated with curettage (the mean time to healing of the wound for the excision group was 3.6 ± 1.2 weeks vs. 11.4 ± 5.1 weeks for the curettage group). Postoperative transient marginal mandibular nerve weakness of the facial nerve was seen in four patients in the excision group, whereas no facial nerve problems were observed in the curettage group [69].

Several other retrospective studies or non-interventional prospective studies have evaluated the treatment and the management of NTM lymphadenitis. In a retrospective study, Luong et al. showed that in some cases, treatment with antibiotics alone is successful and adjuvant therapy to surgical excision is useful to achieved complete resolution of the lymphadenitis [70].

In a 2-year prospective surveillance study, which included 61 children with NTM infections, a conservative approach was followed in a minority (11%) of the patients. Most cases were treated with chemotherapy, surgery, or a combination of both. The resolution was achieved in 39% of patients with chemotherapy alone [46].

In an observational study that included 92 immunocompetent children with NTM lymphadenitis (with positive culture obtained by fine-needle aspiration), Zeharia et al. showed that the observational approach could be effective for managing NTM lymphadenitis [48]. The total resolution was achieved within 3–6 months in 65 (71%) patients, within 9 months in 25 (27%), and within 12 months in 2 (2%). There were no recurrences [48].

In a retrospective cohort study of NTM cases over a 10-year-period at a tertiary referral hospital in Australia, which included 107 children with NTM lymphadenitis, it was found that anti-mycobacterial combination therapy was associated with a reduced risk of recurrences in patients with NTM lymphadenitis compared with cases which received clarithromycin only or no anti-mycobacterial treatment [55].

In a recent systematic literature review and meta-analysis performed by Zimmermann et al., 1951 children with NTM lymphadenitis were evaluated, and different treatment modalities were compared (i.e., complete excision, anti-mycobacterial antibiotics, and ‘no intervention’) [34]. Only complete excision showed a significantly higher probability of cure than no intervention (odds ratio 33.3; *p* < 0.0001) [34].

In summary, in light of current evidence, complete surgical excision is correlated with a greater chance of isolating the causative organism, higher cure rates, faster resolution times, fewer recurrences, and improved esthetic results. For these reasons, it remains the best treatment option for NTM cervicofacial lymphadenitis. However, this treatment modality is associated with adverse events, particularly with the highest risk of developing facial palsy. Therefore, treatment decisions should be guided by the certainty of the diagnosis, the location and extent of the disease, and the parent’s compliance towards prolonged use of antibiotics, as well as towards a no intervention approach, correlated with a slow resolution of the disease. When it is not possible to proceed with radical surgical treatment, it is recommended to undertake antibiotic therapy with clarithromycin (15 mg/kg/day in two doses) in combination with rifampicin (10–20 mg/kg in 1 daily dose), rifabutin (5 mg/kg in one dose), or combined with ethambutol (20 mg/kg in 1 daily dose), for a duration of two months [42]. Although further studies on the optimal regimen are needed, ciprofloxacin has shown activity against some atypical mycobacteria and could be considered among the potential drugs for NTM treatment [42].

### 7.2. Cat Scratch Disease

*B. hensalae* is a slow-growing, Gram-negative bacillus responsible for regional lymphadenitis with fever commonly known as cat scratch disease (CSD) [71]. Studying the sequence of the 16S rRNA gene, two main genotypes of *B. henselae* have been discovered in human patients or cats: the Houston-1 serotype and Marseille serotype [71,72]. Bartonella’s infection has a worldwide distribution and affects both the adult population and the pediatric population; although it has a clear prevalence in the pediatric population and in the regions with a temperate climate, it has a greater incidence in the autumn and winter seasons [73]. A recent American study, conducted by Raynolds et al., analyzed approximately 670 cases of pediatric CSD, highlighting a clear prevalence of the disease in the southern regions of America and in the age group between 5 and 17 years, although the incidence rate of hospitalization in children under the age of 5 was higher [74].

Although the pathogenetic mechanism underlying the development of the disease is still not fully explained, it is known that in humans, *B. hensalae* affects endothelial cells triggering a proinflammatory response, which leads to a local infection that manifests in immunocompetent patients as regional lymphadenopathy [75].

The infection is transmitted by direct inoculation through the scratch or bite of the reservoir, typically cats, although exposure to dogs and flea bites have also been linked to this infection. No evidence of person-to-person transmission exists [76]. Few days after inoculation, a papule or a blister appears on the wound site, which then evolves in two or three days first into a vesicular, erythematous, and then papular phase. The primary lesion lasts from one to three weeks. Two weeks after enlarged lymph nodes appear next to the inoculation site, the involved lymph nodes are initially elastic, mobile, tender with typical size from 1 to 5 cm and erythema of the overlying skin. Subsequently, in about 10–15% of the cases, the adenopathy evolves in a suppurative phase, which can last for months. The anatomical regions most involved in over 90% of patients are cervical, axillary, supraclavicular, or epitochanteric [77]. In more than 80% of all cases in immunocompetent patients, the most common presentation is self-limiting regional lymphadenitis persisting for 3 weeks or more without sequelae [78]. Nonetheless, *B. henselae* infection has historically been associated also with visceral, neurological and ocular manifestations [79].

In most cases, the diagnosis of CSD is clinical and supported by a history of exposure to a cat; serologic testing can be used to confirm the diagnosis. Margileth proposed for the diagnosis of CSD the following criteria (3/4 criteria confirm the diagnosis and in an atypical case, all four criteria may be needed) [80]: (1) cat or flea contact with or without a scratch mark or a regional inoculation lesion; (2) negative TST, negative serology for other infectious causes of adenopathy, and sterile pus aspirated from the node, positive PCR assay; CT scan: liver/spleen abscesses; (3) positive serology test >1:64 for *B. henselae* or *B. quintana* or *Bartonella clarridgeiae*; (4) biopsy of skin, node, bone, liver, or eye granuloma showing granulomatous inflammation compatible with cat-scratch disease or positive Warthin-Starry silver stain. It is important to note that the IgM response to *B. henselae* is brief and could be missed. For this reason, a negative result for IgM antibodies may be expected in the course of the illness. However, most patients have elevated IgG antibody titers at presentation. An IgG titer of >1:256 is consistent with acute infection. Low IgG antibody titers might correlate with the onset or the end of the infection but also with prior exposure to *B. henselae.* Therefore, in cases of IgG titers between 1:64 and 1:256, a second serum sample in two weeks is suggested to detect a titer increase, which should confirm the diagnosis [81,82] The positive PCR hybridization assay for *Bartonella* sp. on abscess aspirates or lymph node biopsy has the highest diagnostic sensitivity [80].

Although radiological investigations show non-specific images of lymphadenopathy, they can be useful for differential diagnosis given the wide spectrum of clinical conditions. CSD lymphadenopathy is represented with enlarged lymph nodes with a central area of necrosis and significant edema in the lymphatic drainage area near the site of inoculation [83,84].

Although CSD in immunocompetent persons is usually a self-limited disease, in some patients, the lymph nodes may be painful and have a protracted course with the formation of abscess and fistulas. In these cases, several studies have highlighted the need for antibiotic therapy or even multiple drainages [85,86].

CSD lymphadenopathy tends to regress spontaneously between two and four months even without specific anti-infective treatment. Therefore, in the case of mild symptoms, only symptomatic therapy and follow-up are recommended [42]. However, antimicrobial therapy may shorten the period of symptomatic illness and may promote recovery, particularly in the case of complications associated with infection. Moreover, antibiotic therapy is recommended in visceral bartonellosis [79]. If a suppurative process develops, evacuative aspiration is recommended, which frees the patient from symptoms in 24–48 h [78]. In case of recurrence of suppuration, aspiration is recommended again, while incision and drainage placement are not recommended as they could cause the formation of a chronic fistulation (6 to 13 months) [78]. Table 4 shows the main studies on treatment and management of CSD lymphadenitis in pediatrics [58,85,87,88,89].

In patients with moderate to severe CSD, the practice guidelines for the treatment of CSD, the Infectious Diseases Society of America (IDSA) [86], and the Italian guidelines [42] suggest oral therapy with azithromycin. No other antibiotics have been mentioned as a valid alternative until now.

In an uncontrolled retrospective study of 268 patients with CSD, Margileth et al. showed that only 4 of 18 different antimicrobials were effective in improving clinical findings, such as reduced or resolved lymphadenopathy and declined erythrocyte sedimentation rate [87]. Particularly, the efficacy of three oral drugs in decreasing order was: rifampin 87%, ciprofloxacin 84%, and trimethoprim/sulfamethoxazole (TMP/SMX) 58%. Gentamicin intramuscular was effective in 73% [87].

One small prospective placebo-controlled trial conducted in 29 immunocompetent patients with CSD lymphadenitis showed a significant difference in resolution of lymphadenopathy (measured by ultrasound) with azithromycin compared to placebo, particularly 30 days after initiation of therapy [88].

A recent French retrospective study analyzed 51 patients with suppurated CSD lymphadenitis treated with oral azithromycin combined or not with an intra-nodal injection of gentamicin [85]. Combined treatment was associated with a higher probability of cure by comparison with patients treated with oral azithromycin only (64% versus 31%, *p* = 0.01) [85].

Another retrospective study including 175 children with CSD lymphadenitis showed no statistical difference in the effectiveness based on the resolution or improvement of lymphadenopathy between azithromycin or TMP/SMX therapy, suggesting TMP/SMX as a reasonable alternative to azithromycin [89].

In summary, a typical course of CSD lymphadenitis in immunocompetent children is of a self-limited disease with a slow resolution that occurs in 1–3 months with or without treatment. However, antimicrobial treatment may be effective in prompting lymphadenopathy resolution, particularly in immunocompromised patients or in case of complications, such as suppurated lymphadenitis or fistulation.

### 7.3. Other Infectious Agents

Other infectious causes for persistent lymphadenitis in children are tuberculosis, toxoplasmosis, and sarcoidosis [42]. Moreover, it is important to exclude malignant neoplasm such as Hodgkin’s lymphoma, particularly when a definitive diagnosis is not determined [42].

## 8. Conclusions

Although lymph node enlargement in children is usually benign and self-limited, it is important to exclude malignant etiology. In neonates, lymphadenopathy could suggest vertically transmitted infection (i.e., toxoplasmosis, CMV, HIV) or primary immunodeficiencies [42]. The differential diagnosis for lymphadenopathy in pediatrics after the neonatal period is broad [42]. In most cases, history and physical examination allow to identify the correct diagnosis and start a proper treatment with a prompt resolution of the lymphadenopathy. However, particularly in the case of persistent lymphadenitis, determining the cause of lymph node enlargement may be difficult, and the exact etiology may not be identified, despite extensive investigations. Moreover, there is still no consensus for a definitive approach to the management of this condition. Further studies should develop and validate an algorithm to assist pediatricians in the diagnosis and timely treatment of lymphadenitis in clinical practice, suggesting situations in which a watchful waiting may be considered a safe approach, those in which empiric antibiotic therapy should be administered, and those requiring a timely diagnostic work-up.

## Figures and Tables

**Table 1:** Main zoonosis associated with acute lymphadenitis in pediatric age.

Disease	Agent	Animal	Inoculation
Cat scratch disease	*Bartonella henselae*	Cat	Skin scratch
Toxoplasmosis	*Toxoplasma gondii*	Cat	Ingestion of material contaminated with cat stools
Tularemia	*Francisella tularensis*	Rodents and insects	Tick bite or skin/mucosa exposure to infected animal
Brucellosis	*Brucella* spp.	Sheep, goat, pigs, cattle	Ingestion of unpasteurized milk or contact with animal fluids
Histoplasmosis	*Histoplasma capsulatum*	Birds and bats	Inhalation
Trypanosomiasis	*Trypanosoma brucei*	Tsetse fly	Bite by tsetse fly

**Table 2:** Lymph nodes and their drainage area.

Lymph Node Group	Drainage
Occipital	Posterior scalp
Posterior auricular	Temporal and parietal scalp
Preauricular	Anterior and temporal scalp, anterior ear canal, conjunctiva
Parotid	Midface, middle ear, parotid gland
Jugulodigastric	Tonsillar
Submandibular	Cheek, nose, lips, tongue, gums, buccal mucosa
Submental	Lower lip, floor of mouth, anterior tongue
Superficial cervical	Lower ear and parotid, lower larynx, lower ear canal
Deep cervical	Occipital scalp and posterior neck, ear, tongue, trachea, nasopharynx, thyroid, palate, nose, esophagus, paranasal sinuses
Supraclavicular	Right side: mediastinum, lungs Left side: abdomen
Deltopectoral	Arms
Axillary	Arm, breast, thorax, neck
Epitrochlear	Medial side of arm below elbow
Inguinal	Lower extremity, genitalia, buttock, abdominal wall below umbilicus
Popliteal	Lower leg

**Table 3:** Main studies on the management of nontuberculous mycobacteria (NTM) lymphadenitis in pediatric age.

Authors (Year)	Type of Study	Study Population	Age Median Months (Range)	Therapy	Results
Haverkamp MH (2004) [46]	Prospective study	61 children with NTM infections (92% with lymphadenitis)	31 (6–151)	Treatment modality: 7 (12%) wait and see 24 (39%) medication only 17 (28%) complete surgical excision 13 (21%) medication and surgery	Resolution was achieved in 39% of patients with chemotherapy alone Children with positive culture results did not differ in disease characteristics from those without positive culture results
Luong A et al. (2005) [70]	Retrospective study	55 children with diagnosis of NTM lymphadenitis	21.6 (15–192)	Treatment modality: 30 (54.5%) antibiotics alone (group 1) 15 (27.2%) antibiotics followed by surgical excision (group 2) 5 (9%) surgical excision followed by antibiotics (group 3) 5 (9%) surgical excision alone (group 4)	Resolution occurred in 30/45 (67%) patients who were initially treated with medical therapy Time of response in group 1 patients: 13/15 (87%) children who started on a regimen with clarithromycin alone responded by 2 months of treatment, and resolution occurred in 15/15 (100%) by 6 months Resolution by 6 months occurred in 14/15 (93.3%) children who received clarithromycin + other antibiotics/other antibiotics Time of response in group 2 patients: 6/15 (40%) patients who initially received antibiotics followed by surgical excision responded well to 2 months of treatment 3/10 (30%) children who underwent surgical excision initially (groups 3 and 4) required additional treatment for recurrence
Lindeboom AJ (2007) [66]	RCT	100 children with diagnosis of NTM lymphadenitis	45.5 (9–168)	50 had surgical excision 50 were treated with clarithromycin (15 mg/kg bid) + rifabutin (5 mg/kg qd) both for a period of at least 12 weeks	Surgical excision more effective than antibiotic therapy (96% vs. 66%, IC for the difference 16–44%) Complication of surgery: 14 of 50 patients (28%). Postoperative weakness of the marginal branch of the facial nerve was observed in 7 patients (14%; in 1 patient, it was permanent) 39 (78%) of 50 patients allocated to antibiotic therapy reported adverse effects
Zeharia A (2008) [48]	Observational study	92 children with NTM lymphadenitis (positive culture)	18 (8–13)	Observation alone Follow-up for at least 2 years	Total resolution achieved within: 3–6 months: 65 (71%) patients 9 months: 25 (27%) patients 12 months: 2 (2%) patients There were no recurrences
Lindeboom AJ (2009) [67]	RCT	100 children with diagnosis of NTM lymphadenitis	45.5 (9–168)	50 had surgical excision 50 were treated with clarithromycin (15 mg/kg bid) + rifabutin (5 mg/kg qd) both for a period of at least 12 weeks	Successful surgery group (surgical wound healed completely at the 3- and 6-month evaluation): 48 of 50 (96%) Successful antibiotic group: 33 of 50 (66%) Median OSAS scores of the surgical group (30.6 points) vs. the antibiotics group (42.2 points, Mann–Whitney *U* test, Z = 2.78, *p =* 0.005)
Lindeboom AJ (2011) [68]	RCT	50 children with microbiologically proven NTM lymphadenitis	35 (14–114)	25 received clarithromycin (15 mg/kg bid) + rifabutin (5 mg/kg qd) for 12 weeks 25 received no antibiotic therapy (wait-and-see)	No difference in the time to resolution (median time: 36 weeks clarithromycin + rifabutin antibiotic treatment. vs. 40 weeks wait-and-see policy)
Lindeboom AJ (2012) [69]	RCT	50 children with microbiologically proven NTM lymphadenitis	36 (14–120)	25 treated with surgical excision 25 treated with surgical curettage	The mean time to healing of the wound for the excision group was 3.6 ± 1.2 weeks vs. 11.4 ± 5.1 weeks for the curettage group (*p* < 0.05) Postoperative transient marginal mandibular nerve weakness of the facial nerve was seen in 4 patients in the excision group. No facial nerve problems were observed in the curettage group
Zimmermann P (2015) [46]	Systematic review	1951 children with NTM lymphadenitis from 60 publications	40.8	Treatment modality: 1077 (55%) complete excision 15 (<1%) incomplete excision 121 (6%) curettage 246 (13%) incision and drainage 32 (2%) fine-needle aspiration 87 (5%) other surgery 171 (9%) anti-mycobacterial antibiotics 157 (8%) no intervention	Adjusted mean cure rate: 98% (95% CI 97.0 e 99.5%) for complete excision 73.1% (95% CI 49.6–88.3%) for anti-mycobacterial antibiotics 70.4% (95% CI 49.6–88.3%) for ‘no intervention’ Compared to ‘no intervention’, only complete excision was significantly associated with cure (OR 33.1; *p* < 0.001) Complete excision was associated with a 10% risk of facial nerve palsy (2% permanent)
Tebruegge M (2016) [57]	Retrospective study	107 children with NTM lymphadenitis	31.2 (25.2–45.6)	Treatment modality: 104 (97.2%) complete excision 2 (1.9%) diagnostic biopsies 1 (0.9%) partial excision 14 (13%) course of antimycobacterial treatment post-operatively (for 2–6 months) with: clarithromycin alone (42.9%); clarithromycin + rifampicin (57.1%)	Symptomatic cure at 6 months: 107/107 (100%) Recurrence: 19/107 (17.8%) 28.5% of patients who received anti-mycobacterial drugs vs. 16,1% of patients who did not receive anti-mycobacterial treatment, *p* = 0.2687 ≥2 recurrences in 0/8 cases (0%) treated with clarithromycin and rifampicin versus 7/99 cases (7.1%) without anti-mycobacterial treatment or clarithromycin only; OR 0.73; *p* = 0.6053 Facial palsy as complication of surgery: 8/104 (7.5%)

bid—twice daily; CI—confidence interval; OR—odds ratio; OSAS—Observer Scar Assessment Scale; qd—once a day; RCT —randomized controlled trial.

**Table 4:** Main studies on treatment and management of cat scratch disease (CSD) lymphadenitis.

Authors (Year)	Type of Study	Study Population	Age Median Months (Range)	Therapy	Results
Margileth (1992) [87]	Retrospective study	268 patients with moderate–severe CSD	240 (6–864)	Group 1 (66 patients): no antibiotic Group 2 (113 patients): antibiotic no effective Group 3 (89 patients): antibiotic effective	4/18 different antimicrobials had demonstrable efficacy Antibiotic effectiveness: Rifampin: 13/15 (87%) Ciprofloxacin: 27/32 (84%) Gentamicin: 11/15 (73%) Trimethoprim and sulfamethoxazole: 26/45 (58%) Penicillins, cephalosporins, tetracycline, and erythromycin had minimal or no clinical efficacy
Bass (1998) [88]	RCT	29 children with cat scratch lymphadenopathy	210 (12–670)	15 received oral azithromycin: ≥45.5 kg: single dose of 500 mg on the 1st treatment day, followed by 250 mg daily for treatment days 2–5 <45.5 kg: 10 mg/kg the first day, followed by 5 mg/kg/d for the 2–5 day of treatment 14 received placebo	30 days after initiation of therapy assessment, significative reduction (≥80%) in affected lymph node volume: 7/14 azithromycin group vs. 1/15 placebo group (*p* = 0.026)
Garnier (2016) [85]	Retrospective study	51 patients with suppurated CSD’s lymphadenitis treated with oral azithromycin	Mean age 26.3 years 17/51 (33%) < 15 years	Group 1: 26 (51%) oral azithromycin without intranodal injection of gentamicin Group 2: 25 (49%) received intranodal injection of gentamicin	Combined treatment was related to a higher probability of cure without complication vs. treatment with oral azithromycin only (64% versus 31%, *p* = 0.01) Complication: Group 1: 18/26 (69%), of whom 5 required surgery Group 2: 9/25 (36%), of whom 4 required surgery
Lindeboom (2015) [58]	Prospective study	53 children with cervical lymphadenitis caused by *B. henselae*	59 (16–148)	The patients were not treated with antibiotics 11/51 (21%): repeated aspiration of pus was performed 40/51 (79%): wait-and-see-policy	Mean resolution time: 5 ± 3.1 months in intervention group vs. 8.2 ± 3.8 months in wait-and-see group (*p* = 0.01)
Shorbatli (2018) [89]	Retro-spective study	175 children with CSD lymphadenitis	Mean age 7.4 years	Group 1: 102/175 were treated with oral azithromycin (10 mg/kg/die with maximum of 500 mg orally for day 1 and 5 mg/kg with maximum of 250 mg once daily on days 2–5 as a suspension) Group 2: 18/175 were treated with oral TMP/SMX (trimethoprim component 8–20 mg/kg orally divided twice daily for 7–14 days as a suspension) Group 3: 10/175 received no antibiotic therapy Group 4: 45/175 received single or combined therapy with clindamycin, amoxicillin/clavulanate, doxycycline, cephalexin, ciprofloxacin, erythromycin, incision, and drainage or excision of lymph node	In Group 1, resolution or improvement was achieved in 51.4% (37/72) of patients without additional medical or surgical intervention 48.6% (35/72) not improved: 2 had no additional therapy 33 received a second course of azithromycin, TMP/SMX, erythromycin, amoxicillin/clavulanate, or rifampin with/without surgical intervention. Response to additional interventions was achieved in 78.7% (26/33) In Group 2, resolution or improvement was achieved in 61.5% (8/13) of patients without additional medical or surgical intervention No statistically significant difference in the effectiveness based on CSD resolution or improvement between azithromycin and TMP/SMX groups (*p* = 0.56) (OR 0.66; 95% CI of OR [0.15, 2.56])

CI—confidence interval; OR—odds ratio; TMP/SMX—trimethoprim/sulfamethoxazole.

## Data Availability

Not applicable for a review article.
